# Prognostic Significance of HER3 Expression in Patients with Cervical Cancer

**DOI:** 10.3390/cancers14092139

**Published:** 2022-04-25

**Authors:** Chi-Son Chang, Jung In Shim, Sun-Ju Byeon, Eun Jin Lee, Yoo-Young Lee, Tae-Joong Kim, Jeong-Won Lee, Byoung-Gie Kim, Chel Hun Choi

**Affiliations:** 1 Department of Obstetrics and Gynecology, Samsung Medical Center, Sungkyunkwan University School of Medicine, Seoul 06351, Korea; cs.chang@samsung.com (C.-S.C.); jiin.shim@samsung.com (J.I.S.); ej043.lee@samsung.com (E.J.L.); yooyoung.lee@samsung.com (Y.-Y.L.); tj28.kim@samsung.com (T.-J.K.); garden.lee@samsung.com (J.-W.L.); bksong.kim@samsung.com (B.-G.K.); 2 Department of Pathology, Hallym University Dongtan Sacred Heart Hospital, Hwasung 18450, Korea; byeonsunju@hallym.ac.kr

**Keywords:** HER3, human epidermal growth factor receptor, cervical cancer, immunohistochemistry, prognosis

## Abstract

**Simple Summary:**

Human epidermal growth factor receptors (HER) are involved in important signaling pathways such as cell growth, proliferation, and cell death. HER3 overexpression is associated with poor prognosis in various tumors, but prognostic relevance of HER3 in cervical cancer was not studied. We analyzed HER3 expression in cervical cancer tissue using immunohistochemistry (IHC) and compared disease-free survival (DFS) and overall survival (OS) based on HER3 expression. The presence of the HER3 protein was linked to a poor prognosis in cervical cancer. DFS and OS were linked to lymph node metastasis, histology, and HER3 protein expression. HER3 expression was connected to poor DFS and OS in both low- and high-risk groups of cervical cancer patients. We suggest that HER3 IHC testing might be a useful method for recognizing cervical cancer patients who are likely to progress.

**Abstract:**

HER3 has been recognized to have an oncogenic role in various types of cancer. However, its prognostic significance has not been elucidated in cervical cancer. The aim of this study was to investigate the prognostic significance of HER3 expression in cervical cancer using immunohistochemistry (IHC). HER3 immunohistochemical staining was performed on the tumor tissue samples of 336 cervical cancer patients. The association between the clinicopathological characteristics and patient survival analysis was assessed according to HER3 expression. HER3 IHC staining was positive in 31.0% (104/336) of the cervical cancer patients. A higher proportion of adeno-/adenosquamous carcinoma was observed in the HER3-positive group (34.6%) than in the HER3-negative group (18.8%). In survival analysis, HER3 expression was significantly associated with poorer disease-free survival (DFS) and overall survival (OS) (*p <* 0.001 and *p =* 0.002, respectively). Multivariate analysis also indicated that HER3 expression was an independent prognostic factor for DFS (hazard ratio (HR) = 2.58, 95% confidence interval (CI) 1.42–4.67, *p =* 0.002) and OS (HR = 3.21, 95% CI, 1.26–8.14, *p =* 0.014). HER3 protein expression was a poor prognostic factor of survival in patients with cervical cancer. This finding could help to provide individualized management for these patients.

## 1. Introduction

Cervical cancer is the fourth most common malignancy among women worldwide, and the second most prevalent cancer in several developing countries [1,2,3]. Patients with bulky tumors or adenocarcinoma histology have a poor prognosis despite comprehensive screening programs and vaccination against carcinogenic human papillomavirus (HPV) [4,5]. Clinical characteristics such as stage, lymph node metastasis, tumor size, and parametrial involvement have some prognostic value, but they aren’t enough to estimate recurrence and survival properly. As a result, biomarkers such as molecular markers are required, and patient treatment would be much improved if tumor behavior could be consistently anticipated at the time of first diagnosis [6,7].

By binding to the appropriate ligands, human epidermal growth factor receptors (HER or ErbB) are involved in important signaling pathways such as cell growth, proliferation, and cell death [8,9,10,11]. HER1 (EGFR or ErbB1), HER2 (ErbB2), HER3 (ErbB3), and HER4 (ErbB4) are the four members of the HER family [12,13,14]. Overexpression of HER causes an amplification of the receptor tyrosine kinase pathway and the loss of regulatory effects [10,15]; HER overexpression has been linked to malignant potential and poor prognosis in a variety of malignancies [12,16,17,18,19,20,21,22,23]. HER1 and HER2 targeted agents are employed in the treatment of lung, colorectal, and breast cancer in clinical practice [24,25].

Because it lacks intrinsic tyrosine kinase activity and is unable to bind adenosine triphosphate (ATP), the function and therapeutic relevance of HER3 has been underestimated [12,26]. Recent investigations, however, have established that the heterodimerization of HER3 with HER1/HER2/HER4 activates a signaling network that promotes tumor growth and metastasis [27,28,29,30]. As a result, numerous investigations are underway to create a novel anticancer drug that targets HER3 [31]. In addition to the clinical development of anti-HER3 treatment, clinical attention is being paid to the predictive and prognostic significance of HER3 overexpression in malignant solid tumors. HER3 has been studied for its prognostic value in various solid tumors, including breast, gastric colorectal, and head and neck cancer, although the results have been contradictory [22,32,33,34,35,36]. Few studies in cervical cancer found that HER3 expression was linked to recurrence [37,38], suggesting that HER3 could be a valuable diagnostic marker for these patients’ prognosis. However, little is known about the clinical and prognostic importance of HER3 expression in cervical cancer patients. We used immunohistochemistry (IHC) to investigate the prognostic impact of HER3 expression in a well-defined cohort of cervical cancer patients.

## 2. Methods

### 2.1. Patients and Tumor Samples

We reviewed the medical records of patients with cervical cancer who were treated at a single academic tertiary center in Republic of Korea between 2002 and 2009. Patients who previously had any type of treatment, such as radiation or chemotherapy, were excluded. The immunohistochemistry analysis did not include patients with rare histology or an advanced stage with primary radiation therapy. Tissue samples were collected from the patients who had signed informed consent as approved by the Institutional Review Board at Samsung Medical Center, Seoul, Korea (IRB No. 2009-09-002-002 and 2015-07-122).

In all of the patients, the primary treatment was radical hysterectomy with or without pelvic/para-aortic lymph node dissection. Adjuvant radiotherapy (RT) or concomitant chemoradiotherapy (CCRT) was given to patients who had risk factors. Patients were assessed every three months for the first two years following treatment, every six months for the next three years, and then once a year after that. Patients who relapsed within three years after adjuvant chemoradiation were classed as resistant to chemoradiation [39,40]. From the date of surgery to the date of recurrence or the final follow-up appointment, disease-free survival (DFS) was calculated. Overall survival (OS) was calculated from the date of surgery to the time of death, or from the date of last contact in case of living patients.

### 2.2. Tissue Microarray Formation and Immunohistochemistry

Tissue blocks used for routine pathologic examination were used to create a tissue microarray (TMA). In each case, areas with the most representative histology were chosen, and three 0.6 mm cylindrical tissue cores were taken from formalin-fixed, paraffin-embedded (FFPE) tissue blocks. To ensure that tissue sampling was adequate, light microscopy was used to analyze slice from each microarray stained with hematoxylin and eosin.

A standard streptavidin–peroxidase technique was used for HER3 and phosphorylated HER3 (pHER3) immunohistochemical staining on 4 μm sections of the TMA. We used fresh-cut sections from the original TMA blocks to prevent possible antigenicity loss during slide aging. Heat-induced antigen retrieval was performed for 40 min in a pH 8.0 buffer for HER3 and 20 min in a pH 6.0 buffer for pHER3 after deparaffinization with xylene and rehydration with a graded alcohol series. Endogenous peroxidase activity was inhibited for 5 min at room temperature with 3% H_2_O_2_. The sections were incubated with anti-HER3 rabbit monoclonal antibody at a 1:100 dilution for 60 min and with anti-pHER3 rabbit monoclonal antibody at a 1:500 dilution for 15 min. Antibodies used for immunostaining were anti-HER3 (#12708) and anti-pHER3 (Tyr1289, #4791) from Cell Signaling Technology. The Dako EnVision+ Dual Link System-HRP (Dako) and DAB+ (3,3′-diaminobenzidine; Dako) were used to detect the antigen–antibody reaction.

### 2.3. Immunostaining Quantitative Evaluation

The IHC staining was graded separately by two investigators (SJB and CHC) who were unaware of the clinicopathological findings. According to the distribution pattern across the cores, the level of staining was classified as 0 (no staining), 1+ (weak), 2+ (moderate), and 3+ (strong). By multiplying the intensity score (0–3) by the proportion of stained cells, the overall protein expression was computed, yielding a maximum final histoscore of 300. The expression values were dichotomized for the survival analysis using the cutoff values with the greatest discriminative power (histoscore of 57 for HER3 and 1 for pHER3).

### 2.4. In-Silico Analysis of GSE44001

Data from the Gene Expression Omnibus (GEO) were used to investigate the prognostic significance of HER3 expression [41,42]. GSE44001 (http://www.ncbi.nlm.nih.gov/geo/query/acc.cgi?acc=GSE44001, accessed on 5 July 2021) was evaluated in a total of 300 patient samples. Data from the cDNA-mediated annealing, selection, extension, and ligation (DASL) assay were used for the mRNA analysis [41]. For survival analysis, the acquired mRNA expression values were also dichotomized according to the cutoff values with the highest discriminative power (7.61 for HER3 mRNA [ERBB3]).

### 2.5. Statistical Analysis

R software version 4.0.0 was used to conduct statistical analysis. The continuous variables were compared between groups using the Student’s *t*-test or the Mann-Whitney *U* test. The expression values were dichotomized (positive vs. negative) for survival analysis using the cutoff values showing the most discriminative power in the univariate Cox model for DFS. The Kaplan–Meier method was used to predict survival distributions, and the log-rank test was used to examine the relationship between survival and each parameter. To identify the independent predictors of survival, a Cox proportional hazards model was developed. At values of *p <* 0.05, statistical significance was determined to be present.

## 3. Results

### 3.1. Patients’ Clinicopathological Characteristics

Table 1 summarizes the clinicopathological features of 336 patients based on their HER3 and pHER3 protein expression status. Within a mean follow-up time of 54 months (range of 1–143 months), 46 patients developed recurrence and 20 patients died. Follow radical surgery, 165 patients (49.1%) were treated with adjuvant radiation either with or without concurrent chemotherapy. Except for histologic cell type, there were no significant differences according to HER3 expression between the two groups. The HER3-positive group had a larger proportion of adeno-/adenosquamous carcinoma (34.6%) than in the HER3-negative group (18.8%). The expression of pHER3 did not differ between the two groups.

### 3.2. HER3 Expression and Its Prognostic Significance

**Figure 1:**
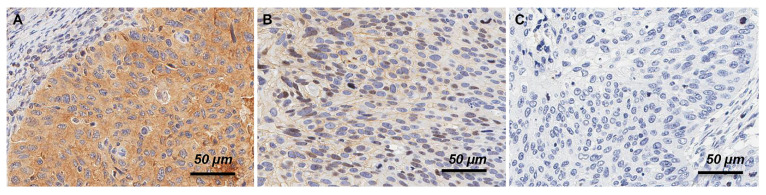
Immunohistochemical expression of (**A**) HER3, (**B**) pHER3 protein, and **(C)** the negative expression of HER3/pHER3 protein in cervical cancer patients.

HER3 and phosphorylated HER3 expression was observed in both the membrane and cytoplasm, and representative examples of positive and negative staining are shown in Figure 1. HER3 was mainly stained in the nucleus, and pHER3 was mainly stained in the cytoplasm. Among the 336 tumors investigated, 104 (31.0%) tumors exhibited positive HER3 protein expression and 22 (6.5%) were positive for pHER3.

**Figure 2:**
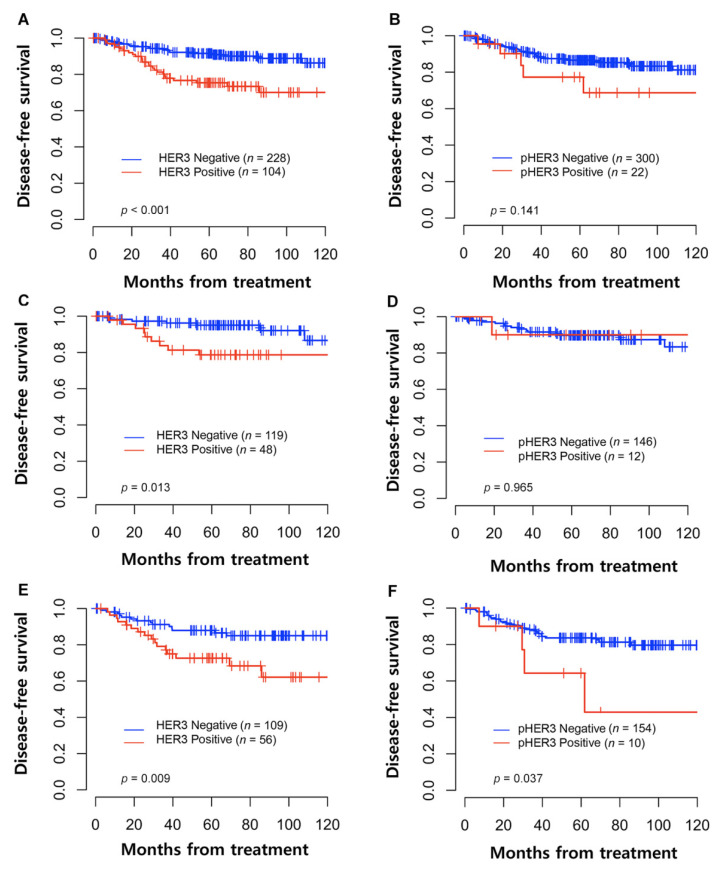
Kaplan–Meier graph illustrating disease-free survival (DFS) according to HER3 and pHER3 expression in patients with cervical cancer. (**A**) DFS according to HER3 expression in total patients, (**B**) DFS according to pHER3 expression in total patients, (**C**) DFS according to HER3 expression in patients without adjuvant treatment, (**D**) DFS according to pHER3 expression in patients without adjuvant treatment, (**E**) DFS according to HER3 expression in patients with adjuvant radiotherapy with or without concurrent chemotherapy, (**F**) DFS according to pHER3 expression in patients with adjuvant radiotherapy with or without concurrent chemotherapy.

The estimated five-year DFS and OS rates for the whole group were 87% (95% confidence interval (CI): 83–91) and 96% (95% CI: 93–98), respectively. The Kaplan–Meier curves for the DFS of cervical cancer patients by HER3 and pHER3 expression are shown in Figure 2. In the total patients, HER3 expression was significantly associated with poorer DFS (*p <* 0.001). Furthermore, we grouped the patients according to whether adjuvant treatment was done. In both groups, the HER3-positive patients showed inferior DFS (Figure 2C,E). Additionally, positive pHER3 protein expression was associated with poor DFS, but was statistically significant only in the group of patients with adjuvant RT with or without concurrent chemotherapy (Figure 2F, *p =* 0.037). Similarly, statistically significant inferior OS was seen in patients with positive HER3 expression (*p =* 0.002), but not in those with positive pHER3 expression (*p =* 0.383) (Figure S1). Based on the clinical significance of HER3 protein expression, the clinical implications of HER3 mRNA expression levels were assessed using data from GSE44001. HER3 mRNA expression levels were also significantly associated with poor DFS (*p =* 0.002) and OS (*p =* 0.017) (Figure S2).

According to the Cox proportional hazards model, HER3 protein expression remained to be an independent prognostic factor for DFS (hazard ratio (HR) = 2.58, 95% CI: 1.42–4.67, *p =* 0.002) and OS (HR = 3.21, 95% CI: 1.26–8.14, *p =* 0.014) (Table 2). However, in multivariate analysis, pHER3 expression was not related with survival.

## 4. Discussion

The present study investigated the prognostic significance of HER3 in cervical cancer by IHC analysis. Inferior DFS and OS were observed in HER3-positive patients, regardless of a high or low risk of recurrence reflected by adjuvant therapy. Furthermore, utilizing data from a prior study [41], consistent results were observed in the examination of the connection between HER3 mRNA and prognosis. These findings imply that HER3 expression could be considered as a prognostic marker in cervical cancer, and that patients with high HER3 expression should become candidates for closer monitoring or more intense adjuvant treatment.

Despite the fact that HER3 has been shown to be overproduced in various types of malignancies [43,44], studies on the relationship between HER3 expression and the prognosis of patients with cervical cancer are scarce. Lee et al. [45] evaluated the mean biomarker expression of HER group for cervical cancer, but HER3 expression was not related to survival outcome. In contrast, another study conducted by Fuchs et al. [37] reported the overexpression of HER3 in 74.4% of squamous cell carcinoma of cervix, and HER3 overexpression was associated with poor OS (90% vs. 69%, *p =* 0.05). In a recent study on cervical adeno-/adenosquamous carcinoma in Japan, 56.7–77.9% showed high HER3 expression with an increased risk of recurrence (HR 6.32, 95% CI: 1.10–36.26, *p =* 0.039) [38]. Our study reinforces the evidence that HER3 expression is associated with the poor prognosis of patients with cervical cancer.

Four members of the HER family form homo- and heterodimers with ligand binding to the receptor. Especially, HER3 has impaired kinase activity and only acquires signaling function when it is dimerized with another HER protein [12]. Therefore, the role of other members of HER family in cervical cancer should also be considered. Recently, Muthusami et al. [46] reported a significant reduction of OS in patients with high EGFR (HER1) expression (HR 0.056, *p* = 0.055). Additionally, poor survival in patients with EGFR overexpression has been reported through a meta-analysis [47]. On the other hand, there are reports with contradictory results showing better prognosis with EGFR expression [37,48]. We performed additional in silico analysis using GSE44001 and TCGA data to examine the clinical significance of EGFR. Our analysis showed significantly better prognosis with expression of EGFR, which can be associated with cell type. As for HER2, multiple reports have produced rather consistent outcomes, showing worse outcome associated with HER2 overexpression [37,40,45]. It is difficult to interpret the results of several studies comprehensively. Many aspects differ from study to study, such as clinical characteristics of patients, method used to detect HER expression, and cut-off values for positive HER expression. Because of this heterogeneity, standardized study design and a larger sample size are necessary to acquire reliable result.

There are many reports about prognostic information of phosphorylated HER2 (pHER2) expression in addition to HER2 status. In patients with HER2-positive primary breast cancer, pHER2-high patients had a lower DFS rate than pHER2-low patients [49,50]. Additionally, correlation of protein expression level between HER2-pHER2 and HER2-pHER3 was reported, supporting that antibodies for pHER2 are a good method for IHC detection of HER2 [51]. However, the prognostic significance of pHER3 in addition to HER3 and correlation between their expression levels has not been evaluated. Unlike HER3 monoclonal antibody, which does not cross-react with other HER family proteins, pHER3 monoclonal antibody may cross-react with overexpressed EGFR and other receptor tyrosine kinases [52]. For this reason, the specificity of pHER3 IHC detection may be interfered with, which is a limitation of this study.

Persistent HPV infections are strongly linked to malignancies of the squamous epithelium, and HPV is thought to be the causal agent in roughly 90% of cervical cancer cases [53]. According to reports in head and neck cancer, there is an association between HPV infection and HER3 expression in HPV-positive tumors [43,54]. Paolini et al. [55] showed that HPV 16 E2 particle may interact with HER3 in conjunction with neuregulin receptor degradation protein 1 (Nrdp-1) in cervical cancer. More research is needed to determine the link between HPV infection and HER3 expression in cervical cancer patients.

Whole-exome or targeted sequencing data including the Cancer Genome Atlas (TCGA) revealed that the incidence of HER3 mutations was higher in adenocarcinoma of the uterine cervix than in squamous cell carcinoma [31,56,57]. These findings are consistent with our results that the HER3-positive group had a greater proportion of adeno-/adenosquamous carcinoma (34.6 vs. 18.8%). The histologic type of adeno-/adenosquamous carcinoma is more resistant to radiotherapy than squamous cell carcinoma [5,58]. Previous study reported that a single dose of radiation induced EGFR expression in cervical cancer tissue, suggesting the role of EGFR in RT resistance [59]. Dittmann et al. have reported that irradiation induced nuclear import of EGFR with T654 phosphorylation, increasing kinase activity essential for DNA repair [60,61]. In this study, pHER3 expression was associated with worse prognosis in patients with adjuvant RT or CCRT (*p* = 0.037). Also, RT resistance rate was higher in pHER3-positive patients than pHER3-negative patients (42.9 vs. 13.6%, *p* = 0.119), but without statistical significance. Only a small proportion of the total cohort (6.5%) exhibited expression of pHER3 in our study. This might imply that resistance to radiotherapy is related to pHER3 expression. However, evidence is still weak, and further study is needed in this regard.

Due to its resistance to radiotherapy, there is a need for novel therapies in patients with adenocarcinoma of the cervix. Studies on HER3 targeting agents are on-going [31], and HER3 inhibition has been proposed as a suppressor of tumorigenesis in head and neck squamous cell carcinoma [62]. Moreover, there is growing evidence for the role of compensatory overexpression of HER3 upon administration of EGFR or HER2 inhibitors, which could be a possible mechanism of resistance to these agents [13]. In HER2-amplified breast cancer, increased level of HER ligands due to treatment with trastuzumab led to an increase in active EGFR/HER3 dimers to promote resistance [63]. For cervical cancer, a French group reported that the combination of EGFR/HER3 dual antibody and chemoradiotherapy enhanced cancer cell death in cervical squamous cell carcinoma cells in vitro and a mouse model experiment [64]. These data indicate that HER3 could potentially be applied as a therapeutic target for cervical cancer.

## 5. Conclusions

Our findings demonstrated that HER3 protein expression was an independent poor prognostic factor of survival in cervical cancer patients. This information may be clinically useful in identifying cervical cancer patients who are at high risk of progression and may be useful in the management of cervical cancer patients.

## Figures and Tables

**Table 1:** The 336 cervical cancer patients’ clinicopathological characteristics.

	HER3			pHER3		
	Negative	Positive	*p*-Value	Negative	Positive	*p*-Value
**Age, years, median [range]**	48.0 [42.0–58.0]	48.0 [41.0–56.0]	0.549	48.0 [41.0–57.0]	47.5 [39.0–56.0]	0.593
**Stage, *n* [%]**			0.406			0.107
IB	200 [87.7%]	87 [83.7%]		256 [85.3%]	22 [100.0%]	
IIB	28 [12.3%]	17 [16.3%]		44 [14.7%]	0 [0.0%]	
**Primary Treatment, *n* [%]**			0.528			0.286
OP only	119 [52.2%]	48 [46.2%]		146 [48.7%]	12 [54.5%]	
OP + RT/CCRT	109 [47.8%]	56 [53.8%]		154 [51.3%]	10 [45.5%]	
**LN Metastasis, *n* [%]**			0.690			0.571
Negative	175 [76.8%]	77 [74.0%]		228 [76.0%]	15 [68.2%]	
Positive	53 [23.2%]	27 [26.0%]		72 [24.0%]	7 [31.8%]	
**Cell type, *n* [%]**			0.003			0.770
SCC	185 [81.1%]	68 [65.4%]		230 [76.7%]	18 [81.8%]	
AD/ASC	43 [18.8%]	36 [34.6%]		70 [23.3%]	4 [18.2%]	
**RT resistance, *n* [%]**			0.056			0.119
Sensitive	79 [89.8%]	34 [75.6%]		108 [86.4%]	4 [57.1%]	
Resistant	9 [10.2%]	11 [24.4%]		17 [13.6%]	3 [42.9%]	
**Tumor size, *n* [%]**	3.0 [2.0–4.0]	3.2 [2.4–4.2]	0.171	3.0 [2.2–4.0]	3.0 [1.2–4.2]	0.357
**PM involvement, *n* [%]**			1.000			0.226
Negative	207 [90.8%]	94 [90.4%]		269 [89.7%]	22 [100.0%]	
Positive	21 [9.2%]	10 [9.6%]		31 [10.3%]	0 [0.0%]	
**Resection margin, *n* [%]**			0.337			0.709
Negative	217 [95.2%]	102 [98.1%]		288 [96.0%]	22 [100.0%]	
Positive	11 [4.8%]	2 [1.9%]		12 [4.0%]	0 [0.0%]	

Abbreviations: OP = operation; RT = radiotherapy; CCRT = concurrent chemoradiotherapy; LN = lymph node; SCC = squamous cell carcinoma; AD = adenocarcinoma; ASC = adenosquamous cell carcinoma; PM = parametrium.

**Table 2:** Multivariate analysis of the relationship between prognostic factors and survival in cervical cancer patients.

Variables	Disease-Free Survival		Overall Survival	
	HR [95% CI]	*p*-Value	HR [95% CI]	*p*-Value
Stage (ⅡB vs. IB)	1.59 [0.78–3.27]	0.202	1.79 [0.61–5.29]	0.290
LN metastasis	4.13 [2.21–7.72]	<0.001	3.02 [1.18–7.74]	0.021
Cell type (AD vs. SCC)	3.05 [1.68–5.54]	<0.001	4.44 [1.76–11.19]	0.002
Tumor size	1.04 [0.86–1.24]	0.707	1.00 [0.77–1.30]	0.991
PM involvement	1.33 [0.57–3.10]	0.503	2.04 [0.60–6.98]	0.256
HER3 (Positive vs. negative)	2.58 [1.42–4.67]	0.002	3.21 [1.26–8.14]	0.014
pHER3 (Positive vs. negative)	1.91 [0.72–5.06]	0.194	2.10 [0.45–9.82]	0.346

Abbreviations: HR = hazard ratio; CI = confidence interval; LN = lymph node; AD = adenocarcinoma; SCC = squamous cell carcinoma; PM = parametrium.

## Data Availability

All data is available in the main text or Supplementary Materials. The RNA-Seq raw data was available at NCBI Gene Expression Omnibus (GEO) (GSE44001). All other data supporting the findings of this study are available from the corresponding author upon reasonable request.

## Supplementary Materials

The following supporting information can be downloaded at: https://www.mdpi.com/article/10.3390/cancers14092139/s1, Figure S1: Kaplan–Meier graph showing overall survival (OS) according to HER3 and pHER3 expression in patients with cervical cancer, Figure S2: Kaplan–Meier graph showing disease-free survival (DFS) and overall survival (OS) according to HER3 mRNA expression in patients with cervical cancer.
