# Supplementary material for: Prognostic Significance of HER3 Expression in Patients with Cervical Cancer
Source: Cancers (Basel). 2022 Apr 25;14(9):2139. doi: 10.3390/cancers14092139 (PMC9104480; doi:10.3390/cancers14092139)
Supplement: Supplementary file 1 [file cancers-14-02139-s001.zip › cancers-1650422-supplementary.pdf]

Supplementary information

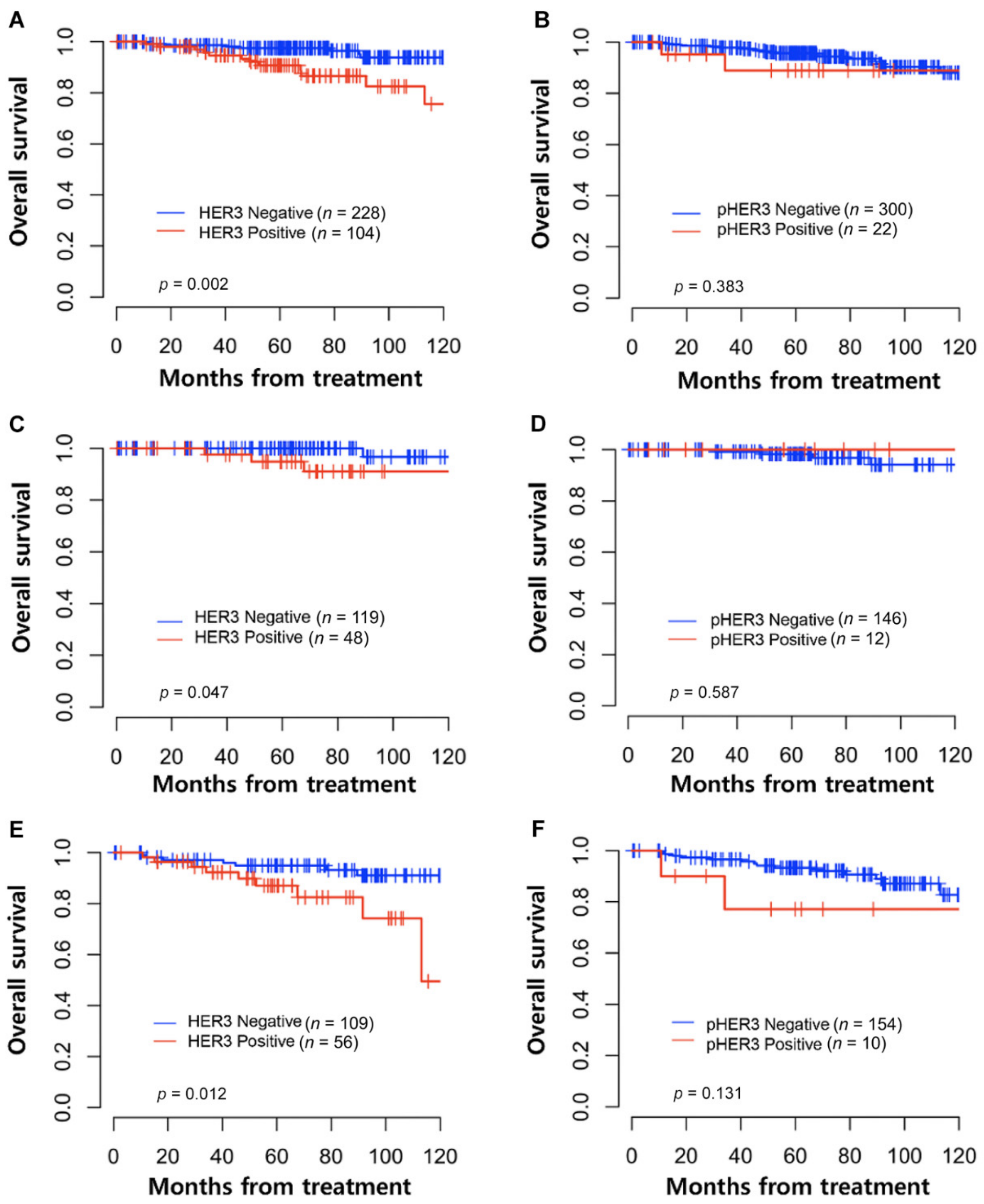

Figure S1. Kaplan-Meier graph showing overall survival (OS) according to HER3 and pHER3 expression in patients with cervical cancer. (A) OS according to HER3 expression in total patients, (B) OS according to pHER3 expression in total patients, (C) OS according to HER3 expression in patients without adjuvant treatment, (D) OS according to pHER3 expression in patients without adjuvant treatment, (E) OS according to HER3 expression in patients with adjuvant radiotherapy with or without concurrent chemotherapy, (F) OS according to pHER3 expression in patients with adjuvant radiotherapy with or without concurrent chemotherapy.

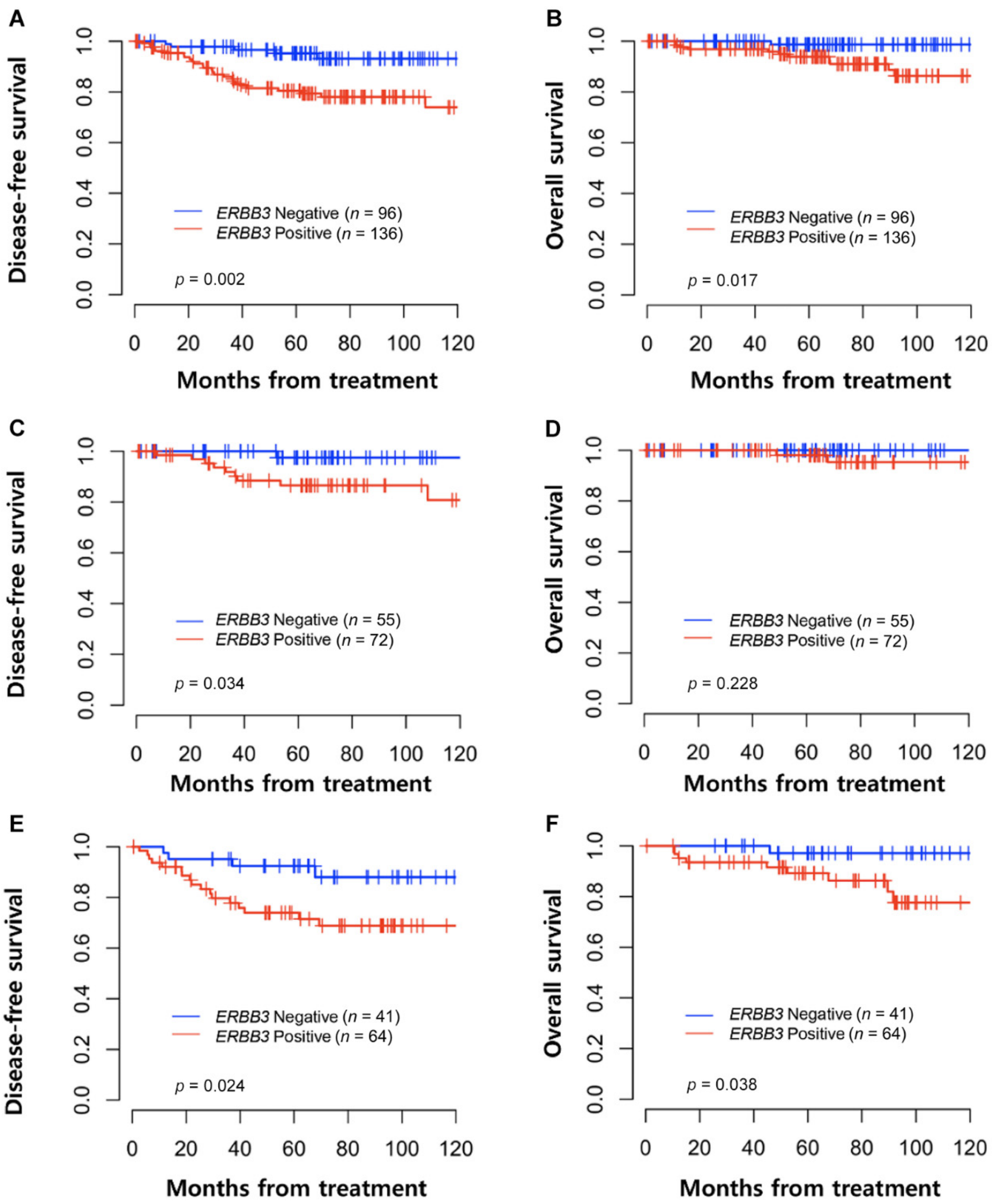

Figure S2. Kaplan-Meier graph showing disease-free survival (DFS) and overall survival (OS) according to HER3 mRNA expression in patients with cervical cancer. (A) DFS according to HER3 mRNA expression in total patients, (B) OS according to HER3 mRNA expression in total patients, (C) DFS according to HER3 mRNA expression in patients without adjuvant treatment, (D) OS according to HER3 mRNA expression in patients without adjuvant treatment, (E) DFS according to HER3 mRNA expression in patients with adjuvant radiotherapy with or without concurrent chemotherapy, (F) OS ccording to HER3 mRNA expression in patients with adjuvant radiotherapy with or without concurrent chemotherapy.
